# Supplementary material for: National Monitoring for Menstrual Health and Hygiene: Is the Type of Menstrual Material Used Indicative of Needs Across 10 Countries?
Source: Int J Environ Res Public Health. 2020 Apr 12;17(8):2633. doi: 10.3390/ijerph17082633 (PMC7215803; doi:10.3390/ijerph17082633)
Supplement: Supplementary file 1 [file ijerph-17-02633-s001.pdf]

Supplementary Materials

Table S1. Reported menstrual material needs by menstrual material use (categorized using both single- and multi-response variables) for each survey

	Uganda				Kenya				Ethiopia			
	None	Absorbent	Money/ vendor	N	None	Absorbent	Money/ vendor	N	None	Absorbent	Money/ vendor	N
**Menstrual Material (Grouped)**												
Pads only	60.35	26.07	13.58	1399	64.51	17.54	17.95	3376	68.36	19.94	11.71	1616
Cloth only	29.71	56.11	14.19	824	20.53	62.04	17.43	392	29.39	57.26	13.35	1836
Cotton wool only	41.94	37.09	20.97	43	54.51	24.79	20.7	110	59.72	24.98	15.31	56
Pads, cloth, cotton wool	37.46	45.43	17.11	339	36.68	36.13	27.19	339	38.51	46.32	15.17	337
Pads, cloth, cotton wool, other	24.81	54.82	20.37	31	27.98	37.55	34.48	98	34.37	56.88	8.74	120
Other[1]	26.49	58.43	15.08	54	47.08	47.89	5.03	42	53.81	33.39	12.8	791
**Menstrual Material (multi-response)**												
Pads	55.98	29.9	14.13	1727	61.74	19.02	19.25	3766	64.84	23.33	11.83	1910
Cloth	30.72	53.61	15.68	1131	22.15	58.69	19.16	617	30.21	56.35	13.44	2262
Cotton wool	43.65	40.03	16.32	137	43.39	26.44	30.17	319	41.88	37.2	20.93	146
Paper	20.68	52.09	27.23	17	37.9	28.92	33.19	97	0	53.46	46.54	9
All others[2]	24.97	60.2	14.83	75	23.44	64.3	12.26	45	51.77	36.31	11.92	903
	**Ghana**				**Burkina Faso**				**Niger**			
**Menstrual Material (grouped)**												
Pads only	80.61	9.03	10.36	2344	63.25	28.65	8.1	290	66.74	20.03	13.23	185
Cloth only	58.14	25.85	16.01	245	31.39	47.73	20.89	1244	51.26	25.37	23.37	1126
Cotton wool only	59.15	40.85	0	8	57.03	26.96	16.01	279	80.52	9.41	10.07	231
Pads, cloth, cotton wool	45.53	18.04	36.44	96	33.86	41.65	24.49	87	43.36	22.08	34.56	35
Pads, cloth, cotton wool, other	60.59	16.39	23.02	98	31.23	51.08	17.69	39	24.56	56.08	19.36	48
Other[1]	62.91	32.52	4.57	44	53.08	39.88	7.04	198	70.2	24.55	5.26	272

	Ghana				Burkina Faso				Niger			
	**None**	**Absorbent**	**Money/ vendor**	**N**	**None**	**Absorbent**	**Money or vendor**	**N**	**None**	**Absorbent**	**Money/ vendor**	**N**
**Menstrual Material (multi-response)**												
Pad/tampon	78.54	9.67	11.79	2533	58.15	31.77	10.08	352	65.97	19.24	14.79	203
Cloth	52.78	24.49	22.73	339	31.24	47.75	21.01	1352	49.92	26.44	23.64	1200
Cotton wool	80.27	11.82	7.91	28	55.01	27.13	17.86	326	75.55	11.88	12.57	262
Paper	62.45	14.31	23.23	93	49.69	43.63	6.68	84	24.87	75.13	0	4
All others[2]	58.67	35.33	6.01	49	49.35	40.68	9.97	153	63.79	28.74	7.47	316
	**Lagos, Nigeria**				**Kaduna, Nigeria**				**Kinshasa, DRC**			
**Menstrual Material (grouped)**												
Pads only	96.47	2.17	1.36	867	77.96	6.66	15.38	542	53.01	31.45	15.54	1525
Cloth Only	76.48	14.58	8.94	46	61.03	15.95	23.02	1090	36.6	39.94	23.47	75
Cotton wool only	76.47	23.53	0	14	70.62	12.93	16.45	6	52.41	44.67	2.93	17
Pads, cloth, cotton wool	83.18	3.46	13.36	29	58.04	13.38	28.58	176	46.77	36	17.23	40
Pads, cloth, cotton wool, other	94.2	4.42	1.38	110	56.8	21.4	21.8	88	16.21	30.36	53.43	230
Other[1]	93.18	4.7	2.12	94	50.97	33	16.03	67	36.19	47.74	16.07	192
**Menstrual Material (multi-response)**												
Pad/tampon	96.04	2.23	1.74	994	73.59	8.37	18.04	743	33.75	43.78	22.47	418
Cloth	76.62	13.58	9.8	76	59.7	16.23	24.07	1327	55.71	29.48	14.82	448
Cotton wool	86.93	10.99	2.08	29	82.12	12.35	5.53	24	44.78	42.61	12.6	189
Paper	94.4	3.82	1.78	197	59.74	26.09	14.17	93	50.87	28.91	20.23	278
All others[2]	74.45	25.55	0	7	46.69	26.7	26.61	63	43.16	35.63	21.21	116

	Kongo Central, DRC				Indonesia				Rajasthan, India			
	**None**	**Absorbent**	**Money/ vendor**	**N**	**None**	**Absorbent**	**Money or vendor**	**N**	**None**	**Absorbent**	**Money/ vendor**	**N**
**Menstrual Material (grouped)**												
Pads only	35.67	37.74	26.59	227	92.23	5.46	2.31	7051	76.21	7.28	16.51	1960
Cloth only	54.66	29.41	15.93	327	89.29	7.45	3.26	585	47.16	21.45	31.39	2456
Cotton wool only	41.57	38.32	20.11	75	100.00	0	0	6	89.27	6.8	3.93	13
Pads, cloth, cotton wool	42.74	48.72	8.53	92	82.26	14.23	3.51	234	57.2	12.97	29.83	519
Pads, cloth, cotton wool, other	39.77	41.03	19.2	171	93.49	6.51	0	13	56.39	28.48	15.13	12
Other[1]	54.44	22.98	22.58	211	90.46	5.15	4.39	145	64.57	20.41	15.03	51
**Menstrual Material (multi-response)**												
Pad/tampon	48.2	31.4	20.4	1778	91.91	5.75	2.34	7297	72.52	8.42	19.05	2469
Cloth	28.54	41.39	30.07	154	87.25	9.44	3.31	824	48.85	20.06	31.08	2978
Cotton wool	54.63	33.33	12.05	47	100	0	0	19	58.6	9.3	32.11	70
Paper	18.63	37.04	44.33	317	100	0	0	12	100	0	0	5
All others[2]	46.33	41.49	12.18	110	89.92	5.71	4.36	146	60.24	23.6	16.17	59

[1] Includes foam, natural materials, bucket, paper, and "other" from the original question, or any combination of those materials, and no materials

[2] Includes foam, natural materials, no materials, bucket, and responses originally recorded as other
